# Improving Just-in-Time Delivery Performance of IoT-Enabled Flexible Manufacturing Systems with AGV Based Material Transportation

**DOI:** 10.3390/s20216333

**Published:** 2020-11-06

**Authors:** Fengjia Yao, Bugra Alkan, Bilal Ahmad, Robert Harrison

**Affiliations:** 1 Warwick Manufacturing Group (WMG), University of Warwick, Coventry CV4 7AL, UK; fengjia.yao@warwick.ac.uk (F.Y.); b.ahmad@warwick.ac.uk (B.A.); robert.harrison@warwick.ac.uk (R.H.); 2 Department of Computer Science and Informatics, London South Bank University, London SE1 0AA, UK

**Keywords:** internet-of-things, flexible manufacturing systems, shop-floor logistics, industry 4.0, autonomous guided vehicles, decision support systems

## Abstract

Autonomous guided vehicles (AGVs) are driverless material handling systems used for transportation of pallets and line side supply of materials to provide flexibility and agility in shop-floor logistics. Scheduling of shop-floor logistics in such systems is a challenging task due to their complex nature associated with the multiple part types and alternate material transfer routings. This paper presents a decision support system capable of supporting shop-floor decision-making activities during the event of manufacturing disruptions by automatically adjusting both AGV and machine schedules in Flexible Manufacturing Systems (FMSs). The proposed system uses discrete event simulation (DES) models enhanced by the Internet-of-Things (IoT) enabled digital integration and employs a nonlinear mixed integer programming Genetic Algorithm (GA) to find near-optimal production schedules prioritising the just-in-time (JIT) material delivery performance and energy efficiency of the material transportation. The performance of the proposed system is tested on the Integrated Manufacturing and Logistics (IML) demonstrator at WMG, University of Warwick. The results showed that the developed system can find the near-optimal solutions for production schedules subjected to production anomalies in a negligible time, thereby supporting shop-floor decision-making activities effectively and rapidly.

## 1. Introduction

In today’s highly competitive and uncertain manufacturing environment, agility and flexibility are two key factors that manufacturing systems need to possess to operate optimally and to adapt to manufacturing disturbances with minimal human intervention. Along with the recent advancements in Industry 4.0 and related technologies, a rapid configuration of manufacturing systems can be achieved through the dynamic planning of shop-floor logistics, real-time optimisation of manufacturing schedules and customised production requirements [1,2,3]. In this context, autonomous guided vehicles (AGVs) became an appropriate enabler to perform versatile jobs in manufacturing shop-floors. In recent years, AGVs are increasingly deployed on shop-floors to replace human labour for material handling and/or transportation jobs with an uncompromised performance [4]. This is due to their ability to help increase the manufacturing efficiency and productivity owing to their flexibility and agility [5].

In Flexible Manufacturing Systems (FMSs) with AGV based material transportation, the due time of AGVs including their earliness and lateness is significantly important in satisfying both the expected overall takt time and production cost [6,7]. Earliness leads AGVs to wait in idle, whereas lateness puts human operators and machines in temporary wait state which results in loss of production [8]. To overcome such a challenge, an optimal dispatch time of AGVs including both start time of operations for jobs at each machine in production stages and precedence relation constraints is required [9]. The previous literature concluded that the efficiency of AGV fleet management highly depends on the selection of dispatching and routing mechanisms as well as the overall integration of the AGV and machine schedules [10]. The overall integration of AGV and machine scheduling dramatically increases the complexity of FMS scheduling, as it does not only involve the job operation sequencing, but also the assignment of material handling tasks to corresponding AGVs by considering the arrival and departure time of vehicles [11,12,13]. This is particularly difficult as a consequence of nature in predicting the AGV transportation times as the conflicts and interferences among AGVs often cannot be neglected. As a result, there is an increasing need for IT tools to schedule/reschedule FMSs based on the integrated machine and AGV operations to rapidly respond to various manufacturing disruptions to operate in an optimal manner [14,15,16].

This paper presents the Smart AGV Management System (SAMS) aiming to integrate real-time shop-floor monitoring and analytics systems with production schedules of machines and AGVs to support shop-floor decision-making activities during the event of manufacturing disruptions. The SAMS and its system architecture were initially proposed in [17]. In this paper, we extend the SAMS architecture by adding a set of novel decision-support capabilities. Towards this aim, an architectural decision-support layer is designed and developed to support shop-floor decision-making at the event of manufacturing disruptions (e.g., machine breakdowns). The SAMS architecture includes a discrete event simulation (DES) model as the digital replica of the FMS under consideration, in which field-level Internet-of-Things (IoT) enabled production data are streamlined and used to enhance the accuracy of the operational behaviours of the entities defined within DES models. In the proposed framework, the production schedule is produced based on both the real-time demand information and resource status information with the help of a Mixed-Integer Nonlinear Programming (MINLP) using Genetic Algorithm (GA) integrated with the DES model. The proposed system can actively sense and transfer production abnormality information to the production management system, such that a rescheduling instruction can be released as a response action. The proposed system is deployed in the Integrated Manufacturing Logistics (IML) demonstrator developed by Automation Systems Group (ASG) at WMG, University of Warwick. The IML is a full-scale FMS integrating logistics with manufacturing operations. This system showcases Industry 4.0 methods, and encompasses both new production systems and legacy equipment within a series of advanced manufacturing scenarios, which is being used for both research and training with a range of industrial partners. The implementation of this research is expected to increase the productivity and flexibility for manufacturing systems by improving shop-floor decision-making efficiency.

The rest of the paper is structured as follows. Section 2 reviews the related literature on the offline and online FMS scheduling approaches and outlines the research gaps. Section 3 presents the overall architecture of the proposed decision support system and data communication protocols. Section 4 details the integrated shop-floor scheduling optimisation approach. Section 5 presents the implementation of the proposed decision support system on the IML demonstrator, and discusses the results and the validity of the approach. Section 6 concludes the paper and outlines the future work.

## 2. Literature Review

In this study, by mainly following the taxonomy proposed by [4], applied methods on the FMS scheduling are grouped into two, i.e., (*i*) offline methods and (*ii*) online (real-time-based) methods. Offline methods are used to schedule FMS operations based on the entire production planning, in which all product components are assumed to be available prior to the start of the production. Online (real-time-based) methods, in contrast, aim at scheduling manufacturing operations at the execution phases, in which shop-floor scheduling decisions are required as the manufacturing system’s status changes. Applied methods on the offline scheduling can be further divided into the following categories: (*i*) the exact methods, (*ii*) heuristics, and (*iii*) simulation-based methods [18]. The exact solution methods aim at achieving the global optimum. [19]. Demesure et al. [20] proposed an AGV navigation approach for FMSs based on the combined use of a motion planner and a priority-based negotiation algorithm. Fontes and Homayouni [21] addressed the integrated scheduling of machines and AGVs in an FMS. In their approach, the FMS scheduling problem is approached using a novel mixed-integer linear programming model, where chained decisions for both machines and AGVs are connected through the completion time-constraints. Fazlollahtabar [22] proposed an AGV scheduling optimisation approach based on the minimum-cost network flow (MCF) algorithm. The approach optimises weighted completion time of tasks for each short-term window by formulating the problem of task and resource assignment as an MCF problem during each short-term scheduling.

Heuristics and meta-heuristics-based search methods are often used in scheduling of FMSs. Dang and Nielsen [23] presented a genetic algorithm-based scheduling optimisation approach for AGV based FMSs. Nageswararao et al. [24] proposed a scheduling approach simultaneously optimising both machine and AGV schedules, based on the implementation of binary particle swarm optimisation approach and vehicles assignment heuristic utilising the rebuts factor maximization function and mean tardiness. Huang et al. [25] proposed an AGV scheduling strategy using both admissible and non-admissible heuristic functions and a production-specific search scheme. The approach is aimed at minimising the makespan and maximising the average machine utilisation and tested on a set of randomly generated FMSs generated using Petri nets. In a similar study, Baruwa and Piera [4] proposed an AGV scheduling strategy evaluating all possible AGV scheduling scenarios without the imposition of a specific dispatching rule. The strategy is based on a hybrid heuristic search method, called any-time layered search (ALS), optimising the AGV schedules based on both the makespan and the exit time of the last job of the system. Sanches et al. [26] propose a simultaneous production schedule optimisation approach for both machines and AGVs using an adaptive genetic algorithm minimising the makespan with low running time. Mehrabian et al. [8] developed a two-objective mathematical programming model, i.e., due dates and processing time, integrating flow shop scheduling and AVG routing in an FMS. The model is studied using two meta-heuristics algorithms, i.e., non-dominated Sorting Genetic Algorithm, and a multi-objective particle swarm optimisation approach. Mousavi et al. [27] proposed a mathematical AGV scheduling model integrated with evolutionary algorithms to optimise the task scheduling of AGVs with the objectives of minimizing makespan and number of AGVs while considering the AGVs’ battery charge. Zhong et al. [28] investigated an integrated scheduling problem of a multi-AGV based system with conflict-free path planning using a Hybrid Genetic Algorithm-Particle Swarm Optimization (HGA-PSO) algorithm. Rahman et al. [29] proposed a meta-heuristics-based scheduling approach to minimise the cycle time and total tardiness in a robotic assembly line with multiple AGVs. Wang et al. [30] aimed at improving energy consumption and production efficiency of AGV transportation using a bi-level heuristic algorithm. Liu et al. [31] proposed a multi-objective mathematical optimisation model based on the combination of two Adaptive Genetic Algorithms (AGA) and a Multi-Adaptive Genetic Algorithm (MAGA).

Online (real-time) scheduling approaches allow manufacturing companies to dynamically schedule their production systems to match the desired customer demands promptly. These approaches are, in general, time-constraint methods in which a limited amount of computation time is provided to generate a set of optimal scheduling solutions [4]. Please note that these methods can be either static or dynamic. Weyns et al. [32] developed a dynamic task assignment protocol, called DynCNET, allowing a flexible task assignment approach that can cope with the operational system dynamics. The proposed protocol is an extension of contract net protocol, CNET (see [33]), allowing AGVs’ task assignments dynamically. Another approach, proposed by Chan et al. [34], is a real-time expert system for scheduling parts in an FMS based on two fuzzy-logic based decision-making/selection rules. Wang et al. [35] proposed a multi-agent-based real-time scheduling architecture, called MARS, for IoT-enabled FMSs. The MARS allows dynamic scheduling based on the coordination of real-time status of AGVs carried out by “*bargaining-game-based negotiation mechanism*” and optimises scheduling targets, such as the makespan, the critical machine workload and the total energy consumption. Zhang et al. [36] developed a cyber-physical system based smart production control model for shop-floor material handling and transportation. TF et al. [37] proposed a reinforcement learning-based method for dynamic multi-AGV flow-shop schedules aiming at minimising both the average job delays and the total makespan. Zhao et al. [38] developed a dynamic scheduling system for multi-AGV based smart factories. Sahin et al. [39] developed a multi-agent-based expert system with agent-to-agent communication and negotiations for simultaneous scheduling of both machines and AGVs in a manufacturing system operating under dynamic manufacturing constraints. Their system is based on the Prometheus methodology (see [40]), and is modelled in the JACK agent-based systems development tool. Xu et al. [41] developed an intelligent logistics scheduling model and execution method for AGVs. Their approach is based on the mode of “request-scheduling-response”, and is integrated with Internet-of-Things (IoT) systems to meet the shop-floor demands in real time. The solution method is based on the combined use of a double-level hybrid genetic algorithm and ant colony optimisation (DLH-GA-ACO).

The literature review showed us that many research works are aiming to optimise FMS production schedules with and without considering production uncertainties and abnormalities such as machine breakdowns and sudden customer demand changes. In general, most of these studies investigate FMS schedules based on a static factory environment, thereby providing offline FMS scheduling approaches. The exact solution approaches can be very promising in finding the global optimum; however, they can be computationally very costly due to the vehicle routing problem being proven to be NP-hard [42]. Heuristics-based can be considered as useful tools; in particular, production performance is the main priority in terms of completion time [43]. Nevertheless, these methods have problems with trapping in local minima and equilibrium attraction. Meta heuristics optimisation algorithms, on the other hand, can be a useful solution for this, as these methods involve mechanisms to avoid getting trapped in local minima. Simulation-based approaches offers what-if analyses that can be used to select the best solution among alternatives. The online (real-time) solution methods are very helpful in solving dynamic AGV routing problems. These methods continuously update the solution space as more information exposed or available in real time. Table 1 summarises the literature review.

## 3. Smart AGV Management System (SAMS)

**Figure 1:**
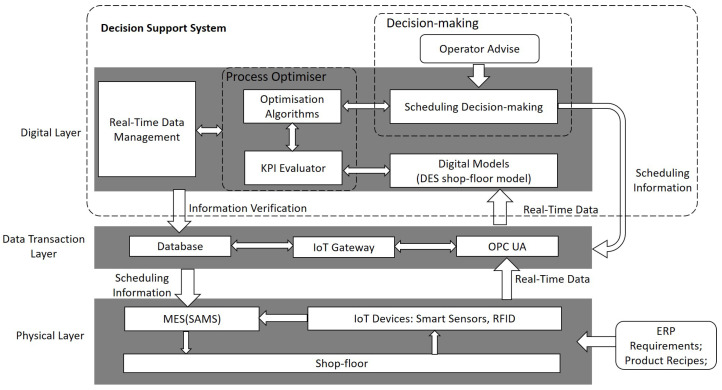
The SAMS architecture.

In this section, the Smart AGV Management System (SAMS) is presented for real-time scheduling optimisation for both AGVs and machines within an FMS. The decision support system connects the Integrated Manufacturing and Logistics (IML) demonstrator rig to DES models, and enables the collection and monitoring of real-time operational information, and prediction and optimisation of the job schedules for: manufacturing processes, and materials delivery and product collection activities. Moreover, the proposed system implements the allocation of AGVs in different workstations, including legacy production loops, standalone autonomous stations and manual operations stations, in shop-floor logistics under the smart factory background. An overview of the SAMS architecture is depicted in Figure 1. In this section, the digital layer of the SAMS is introduced in detail, while other two layers are briefly discussed. Please note that a detailed information about the physical and data-transaction layers can be found in [17].

### 3.1. Physical and Data-Transaction Layers

The bottom two layers of the SAMS are solely responsible for collecting and transferring the real-time production data from IoT-enabled sensing devices in the manufacturing shop-floor to digital layer and vice versa. In the physical layer, two levels of monitoring are considered, i.e., workstation level and system level. Within these monitoring levels, IoT devices, including energy monitor and smart buffer sensors, are implemented on the machines, and three kinds of information, i.e., energy consumption information, machine status information and cycle-time information, are collected from the IoT-enabled field-devices within the production line. This information includes: cycle-time of each machine job, time of each transportation job between two machines, cycle-time for each loading and unloading operation, AGV charging time, AGV energy consumption in each transportation job, status of machines and AGV (i.e., breakdown, run). Cycle time for both stations and system is sensed through the RFID system, whereas machine status information, and products tracking information for different monitoring levels are captured directly from the function blocks (FB) employed within the Programming Logic Controllers (PLCs). Moreover, machine energy consumption information is directly collected from IoT-enabled smart energy meters.

In the SAMS, real-time data sharing between system modules is based on the OPC-UA protocol (see [58,59]). The OPC-UA is a machine to machine (M2M) communication protocol enabling both connectivity and interoperability among different physical and digital components. The real-time data sharing allows the SAMS to monitor and analyse the operational information from shop-floor devices and machines, such as: robots, PLCs, AGVs, and other IoT-enabled field devices, through the industry network. As an example, battery cell buffers based on the IoT-enabled weight scale are monitored, and the quantity of battery cells is updated into OPC-UA server in real time. In addition, battery packs equipped with an RFID tag are tracked by the SAMS to auto-correct the AGVs transporting in real-time. The SAMS database is created in a data transaction layer for storing shop-floor machines and operation data, such as: machine cycle time, AGV energy consumption, and production life-cycle information. The collected data can also be accessed by other supervisory systems for further production key performance indicator (KPI) assessments.

### 3.2. Digital Layer

Manufacturing KPIs are a set of metrics that can be used by manufacturing enterprises to evaluate the success of their manufacturing operations in meeting the performance targets [60]. These metrics include but are not limited to cost, flexibility, energy, (just-in-time) JIT material delivery performance, quality, etc. In the SAMS, the digital layer is mainly developed for the prediction of production KPIs based on a real-time data management system and a DES model coupled with KPI evaluation schemes and heuristics optimisation algorithms.

The real-time data management system is developed as a software plug-in updating operational DES parameters using the real-time production data stored within a time-series database. Currently, the developed system updates the following information within the DES model: (*i*) cycle time information for each manufacturing process, (*ii*) AGV travelling time and (*iii*) AGV energy consumption for each material transfer event, (*iv*) the charging time for each AGV, and (*v*) the demand. Although this approach provides a noticeable increase in prediction accuracy of DES models, it is planned as a future work to replace the real-time data management system with a complex event processing (CEP) engine to provide a better resolution in identifying and anticipating the relationships between the shop-floor events. The DES model uses the historical data captured from the physical layer to define individual operational parameters represented as a probability distribution function (PDF). It also receives the real-time status information of both machines and AGVs from the corresponding PLCs through the OPC-UA connection. Currently, two types of status information are defined, i.e., available and not available. The KPI evaluator sub-module is embedded within the DES Model describing the definitions and algorithms for the real-time production KPIs. These KPIs can be published into a MATLAB optimiser add-on for further evaluation through the OLE Automation Controller communication protocol.

**Figure 2:**
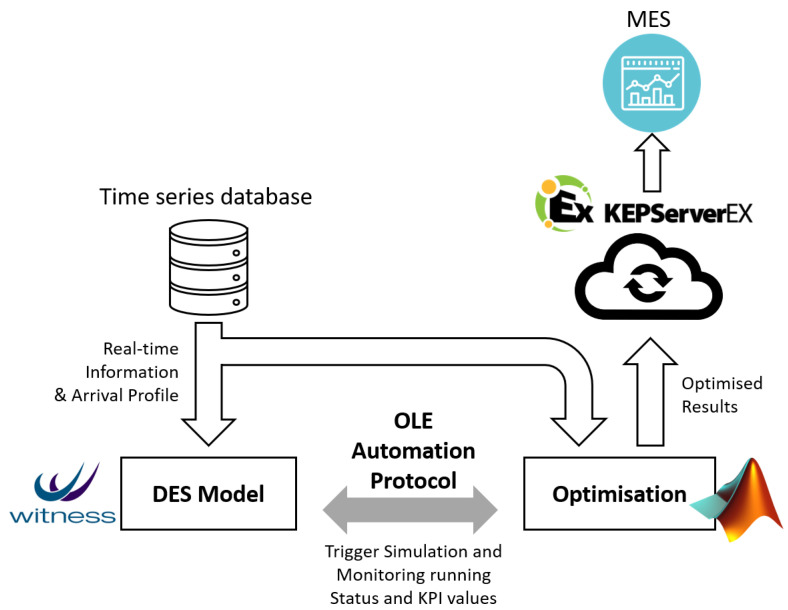
The real-time data communication architecture.

In this research, DES models are built in the WITNESS Simulation Software [61]. The WITNESS DES tool helps engineers to model, analyse and optimise manufacturing processes, so that they can make decisions under a risk-free environment [62]. In general, the WITNESS Simulation Software can build customised manufacturing systems and production processes, and can be connected by external software and databases remotely through WITNESS Command Language (WCL) [63]. It is currently used by various manufacturing companies. For example, Ford UK integrates this software into its assembly line, and has achieved a 10% increase in the production capacity [64]. The WITNESS is capable of generating and analysing production KPIs, such as average material flow time, production cycle time and average AGV energy consumption. In this research, the DES simulations are performed to obtain the production KPIs streamlining into the optimisation engine through OLE Automation Protocol [65]. The OLE Automation Server acts as a data-interface, where commands and the data are transmitted between the WITNESS Simulation Software and the optimisation engine. The communication architecture is depicted in Figure 2.

The optimiser module is responsible for scheduling and re-scheduling both machine and AGV tasks based on evolutionary optimisation algorithms, KPI predictions and real-time resource status information. In the scheduling/rescheduling process, first, the real-time resource status information stored in the time-series database is checked, and the corresponding values are updated within the DES model. Then, a new scheduling instruction is released based on the KPI values obtained from the DES model prioritising the JIT material delivery performance. A mixed-integer Genetic Algorithm (GA) is used in the optimisation of the shop-floor logistics by minimising the JIT error and AGV energy consumption at the same time. Moreover, when a manufacturing disruption occurs, e.g., machine breakdown, the rescheduling mechanism will be triggered to reduce the influence of the disruption, thereby improving the overall production efficiency. In the proposed approach, the decision-making and optimisation modules cooperate to generate the optimal scheduling strategies, and to feed back to the manufacturing execution system (MES) located in the physical-layer. The Decision-making module mainly focuses on the dynamic scheduling strategies under varying production requirements. In such a way, production KPIs predicted by the DES model are evaluated by managers with respect to requirements before being deployed into the MES.

### 3.3. Shop-Floor Decision-Support

**Figure 3:**
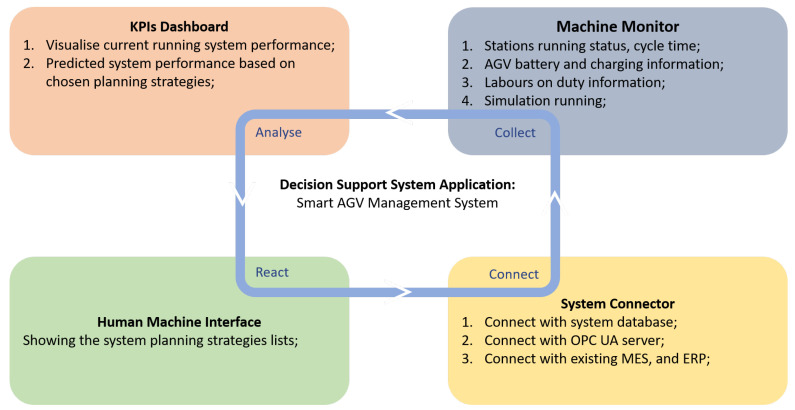
The main components of the decision support system.

The decision support module integrates the SAMS to the existing Products Order System and MES to provide a real-time decision-support functionality during the production process. In the SAMS, the AGV scheduling and production sequences are generated and updated automatically depending on the pre-configured KPI priorities or the manufacturing station change. The integration between the existing systems and the SAMS architecture is done via OPC-UA machine to machine (M2M) communication protocol. The Products Order System used in the experiments is developed by the ASG at WMG, University of Warwick. The implementation details and architecture of this system will be the focus of a future manuscript. The SAMS receives the products order information and customer request updates from the Products Order system and uses this information along with real-time production data to generate a set of production schedules. On the other hand, the OPC-UA connects the SAMS with the MES to monitor the real-time machine states and to track the production processes. The system monitors the real-time production performance, e.g., run-time energy consumption, deviations in process cycle times and overall tardiness. When production abnormalities occur, the SAMS releases a re-scheduling scheme by considering the current machine utilisation and pre-defined KPI targets, such as machines working balance, the average energy consumption and Just-in-Time material delivery performance. The optional scheduling strategies can be chosen by the decision support system about the targeted system KPIs. Alternatively, managers can choose an optimal scheduling strategy through the application HMI and broadcasted KPI dashboards. Once the optimal strategy is selected, the job schedule is sent to the MES system for its execution. Please note that the interoperability of the decision support system allows it to access the system database/server directly. The overview of decision support components of the SAMS is shown in Figure 3.

## 4. Optimisation Approach

In this section, a flow-shop problem is prepared for the IML demonstrator’s factory logistics. The IML is composed of several stages in which machines in the same stage perform identical manufacturing operations. The raw products follow a specific production sequence, and are transported between stages through a number of AGVs. Products are delivered into the packaging area as they are packed as a final product. Please note that each product must go through all production stages one by one in order to finish the entire assembly. It is assumed that every job has a pre-defined due time, and a JIT delivery error occurs if the job is completed after or before its due date (i.e., earliness and lateness). The objective of the problem is to find the near-optimal production schedules including both machines and AGVs that can minimise the total earliness/lateness cost as well as overall energy consumption of AGV operations, simultaneously.

**Figure 4:**
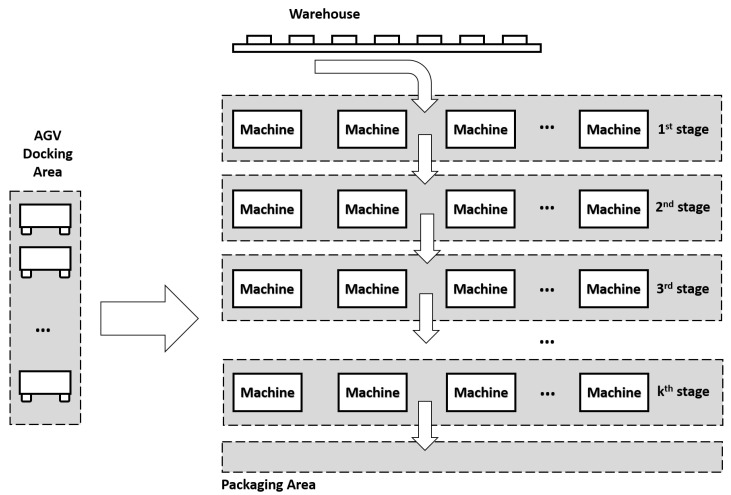
A schematic for the IML shop-floor logistics problem.

A schematic representation of the presented shop-floor logistics problem is given in Figure 4. The IML shop-floor has a tiered flow-shop layout consisting of several stages: including AGV docking area, warehouse, packing area, and work machines area, etc. All AGVs are waiting in the docking area for delivery tasks. Depending on the battery status, AGVs can be recalled back to the docking area for battery recharging. In addition, the AGV parks at the docking station after the completion of the last delivery job if no further jobs are available to the AGV. Raw products are distributed to stations from the warehouse via AGVs, and they are processed through every machine stage until they are delivered to the packing area. These products are transported from one station to another through AGVs based on the delivery schedules generated by the SAMS. AGVs use predefined paths between shop-floor areas, and collisions within each path are continuously monitored and avoided by a supervisory control system.

### 4.1. Problem Formulation

The mathematical notations for the presented shop-floor logistics problems are given in Table 2. The established mathematical model composed of two objective functions, described as follows.

(1) $$Min\left(f\right)=\left\{f_{1},f_{2}\right\}$$

- Objective function 1: aims to minimise the total cost associated with the earliness and lateness of the scheduled jobs, and formulated as below.
  (2) $$f_{1}=\sum_{i=1}^{\left|T\right|}\alpha max\left\{0,d_{i}-C_{i}\right\}+\sum_{i=1}^{\left|T\right|}\beta max\left\{0,C_{i}-d_{i}\right\}$$
  Please note that the authors report based on their project experiences from seat and car manufacturing projects that, overall manufacturing performance, in general, tends to be more affected by the lateness of the jobs. Hence, it is often penalised more than the earliness of the jobs. However, the penalty costs for both earliness and lateness should be configured based on the factory and user requirements.
- Objective function 2: stands for the minimisation of the total energy consumption associated with the AGV loading and cumulative travel distances, and formulated as follows:
  (3) $$\begin{array}{c} f_{2}=\sum_{i=1}^{S}\sum_{j=1}^{S}\sum_{t=1}^{T}\sum_{n=1}^{N^{AGV}}dis_{ij}X_{ijnt}F\left(Q_{n_{o}}+Q_{ijnt}\right) \end{array}$$
  where $F(Q_{n_{o}}+Q_{ijnt})$ represents the energy consumption rate related to AGV weights and travel distance.
- These objectives are subjected to the following constraints:
  (4) $$\begin{array}{cc} \; & S_{ts(w+1)}\geq D_{tsw},\\ & t=1,...,T,\;w=1,...,W,s=1,...,S_{w} \end{array}$$
  (5) $$\begin{array}{cc} \; & S_{tsw}-S_{(t-1)sw}\geq PT_{(t-1)sw},\\ & t=1,...,T,w=1,...,W,s=1,...,S_{w} \end{array}$$
  (6) $$\begin{array}{ccc} \; & max\{\sum_{s=1}^{S_{w}}M_{tsw}\}=1, & t=1,...,T,w=1,...,W \end{array}$$
  (7) $$\begin{array}{ccc} \; & S_{ts1}\geq r_{t}, & t=1,...,T,s=1,...,S_{1} \end{array}$$
  (8) $$\begin{array}{cc} \; & max\{\sum_{t=1}^{T}X_{ijnt}\}=1,\\ & i=1,...,S,j=1,...,S,n=1,...,N^{AGV} \end{array}$$
  (9) $$\begin{array}{c} X_{ijnt},M_{tsw}\in 0,1\\ i=1,...,S,j=1,...,S,s=1,...,S_{w},\\ t=1,...T,w=1,...,W,n=1,...,N^{AGV} \end{array}$$
  In the above equations, constraint (4) is used to ensure that the precedence relations between stages of a job for every AGVs is not breached. Constraint (5) ensures that multiple jobs cannot be performed by a machine at a time. Constraint (6) is used to fulfil the requirement that a job cannot be performed more than one machine in a stage. Constraint (7) enforces the time difference between start time of machine in the first stage and the release time of the jobs that are assigned to them must be equal or greater zero. Constraint (8) ensures that an AGV cannot perform more than one material transportation task at a time. Constraint (9) states the variables’ binary nature.

### 4.2. Assumptions

The following are the assumptions in formulating the model:

- The parameters of machines, including: set up time and processing time are known and based on continuously updated historical production data;
- The parameters of AGVs, including: energy consumption rate, battery capacity and travelling speed are known and based on continuously updated historical production data;
- The demand information is continuously updated in real time;
- Machine output buffers have a fixed capacity limit;
- The AGV fleet capacity is enough to cover all transportation jobs;
- The AGV will not be called by the machine when the machine output buffer is empty.

### 4.3. Genetic Algorithm Based Solving Method

**Figure 5:**
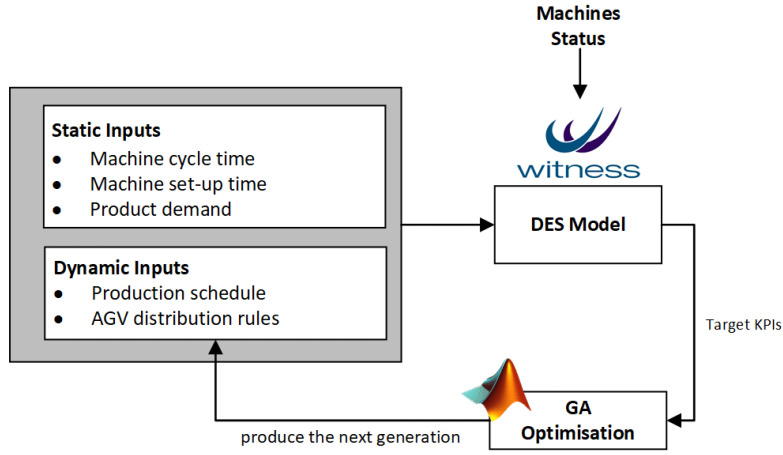
The data-flow between the optimisation module and the DES model.

**Figure 6:**
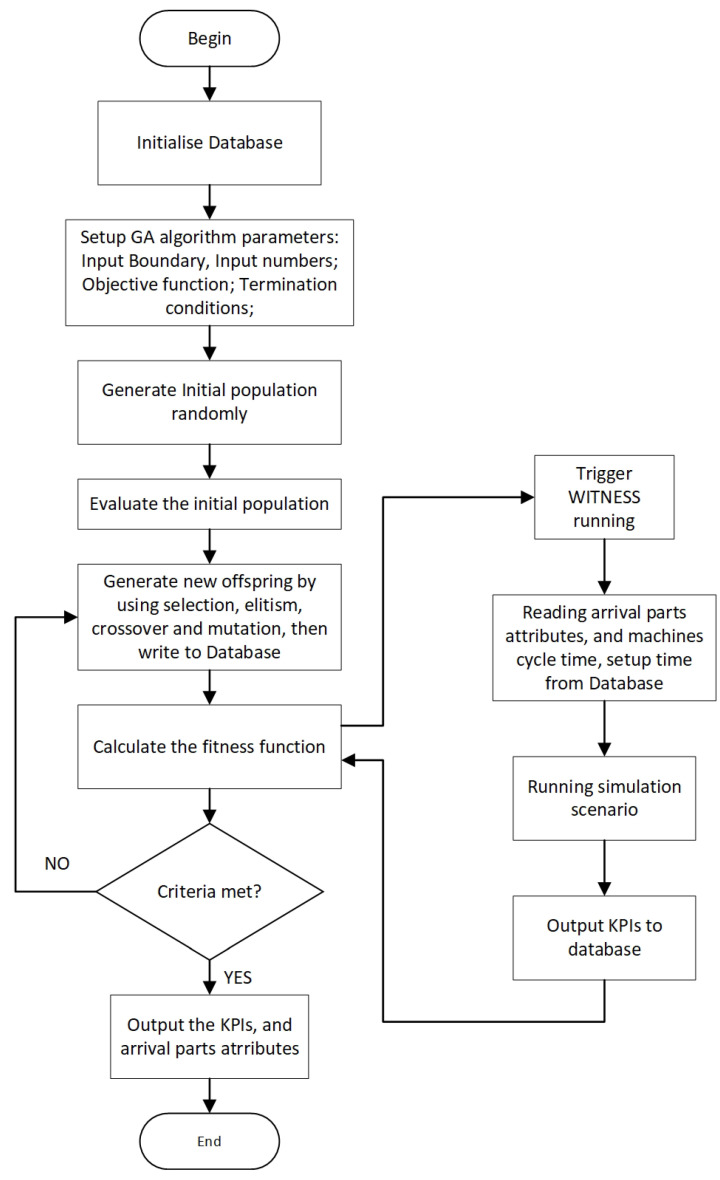
The flow-chart of the approach.

A meta-heuristics algorithm is widely applied for searching the global optimal solution for scheduling problems [66]. In this article, a mixed-integer GA, which is one of evolutionary optimisation algorithms imitating the natural selection and genetics [67], is chosen to search the near-optimal machine jobs sequence and the AGV distribution rules for battery assembly processes performing within the IML. The GA has been used to solve a wide variety of combinatorial optimisation problems and obtained optimal or near-optimal results efficiently. The GA examples for FMS scheduling optimisation problems include: [68,69,70,71,72,73]. The data-flow between optimisation module and the DES model is given in Figure 5. The flow chart of GA-based optimisation approach consisting of the following steps is shown in Figure 6. In the proposed approach, the arrival products sequence and AGV distribution rules for each arrival parts are the input for the DES model, whereas production KPIs are considered as outputs.

### 4.4. Genetic Algorithm

This section presents a GA based optimisation method for AGV and machine jobs schedules in FMSs. The GA method is based on the approach proposed in [74]. In the optimisation approach, fitness function is considered to include: shop-floor processing time, AGV energy consumptions, and machine utilisations mainly derived from the DES simulation. First, a group of initial population is created by the GA algorithm, which are then evaluated through the fitness functions. Following this, a new generation population is created through the selection, crossover, and mutation processes, in which the elitists of current generation are passed to the next populations. The manufacturing processes KPIs: just-in-time performance and cumulative AGV energy consumption are defined as objectives to be improved. The algorithm also stops when the maximum number of generations or number of stall generations are reached. The detail of the GA based optimisation method’s pseudo code is shown in Algorithm 1.    
**Algorithm 1.** Genetic Algorithm pseudo-code.Pseudo-code of the GA1:Initialise the populations;2:Evaluate the initial population through fitness function;3:**for** (iteration < MaxIteration) **do**4: **while** (not meet the stopping criteria) **do**5:  Select the elitists for next generation;6:  Crossover7:  Mutation8:** end while**9: Evaluate the new population fitness;10:**end for**11:Output the best solutions;


#### 4.4.1. Initialising Parameter

**Figure 7:**
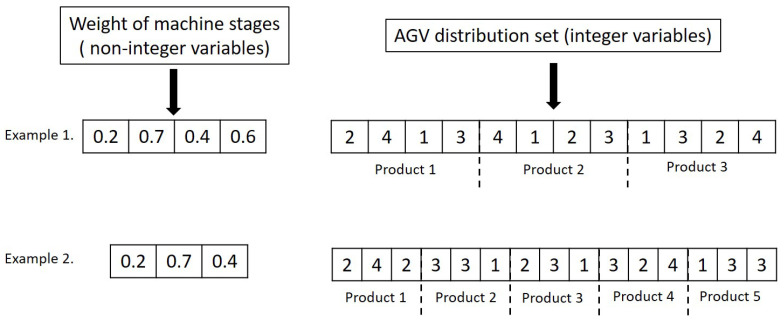
Two examples of the population structure.

In this article, each generation is separated into two segments representing the product sequence and AGV distribution strategies. Figure 7 shows the population structures of two examples. The first example includes a system consisting of three products and four work stages, each having four identical workstations performing operations for three different arrival products, whereas the second example consists of three work stages, five production jobs, and four identical workstations in each stage. The left-hand side in Figure 7, the encoding rule represents non-integer optimisation parameters that are used to define the product sequence to be released from the warehouse. According to this rule, the product sequence is determined based on weighted cumulative cycle times of product variants. This is characterised by cycle times of each product variant at each machine stage and corresponding machine stage weight coefficients. The right-hand side represents the AGV task distribution sequence to be followed by AGVs. This dictates AGVs to transport materials from one stage to another by following the encoding rule.

#### 4.4.2. Initialising Population

The initial population is generated based on the uniform random generator. The first part of variables is in the range of 0 to 1, and the size of their population is considered to be equal with the number of machine stages. In the second part, the size is taken as equal to the product of machine stages number and arrival products number, and the values are limited by the station number in each stage. Therefore, the lower bounds, upper bounds, the number of variables, and the list of integer values are set up to meet these constraints.

#### 4.4.3. The New Generate Population Generating

The new generations are produced by using selection, elitism, crossover, and mutation.

**Figure 8:**
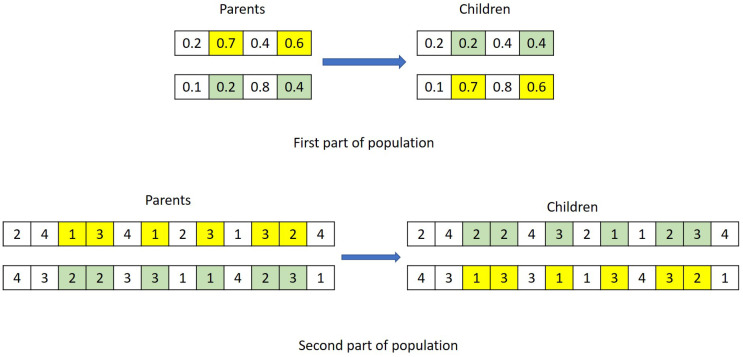
An example of crossover strategy.

**Figure 9:**
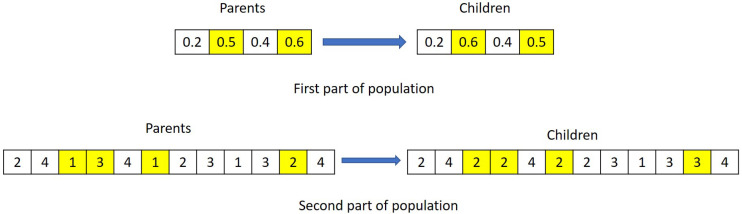
An example of mutation strategy.

- Selection: The stochastic universal selection strategy (see [75]) is used to select parents for producing the next generators. In the stochastic uniform selection, all parents are laid on a line. The algorithm follows the line, and moves to the next point at an equal step size. At each movement, the algorithm chooses the current point as the parent for the next generation. The first step is also a uniform random number, which is smaller than the step size.
- Elitism:All the individuals are sorted based on the fitness values. The first $N_{e}$ (Equation(10)) best individuals are chosen and passed to the next generation directly. This step guarantees that the best fitness values can survive in the next generation:
  (10) $$\begin{array}{c} N_{e}=5\%*PopulationSize \end{array}$$
- Crossover: Crossover is generated by combining the two parents together. The genes from parents are chosen randomly for crossover, and genes coordinates are the same for both parents, and the crossover children population is specified by the crossover fraction $P_{c}$. These rules are applied into both parts of parents. Figure 8 shows an example of crossover strategy.
- Mutation: Mutation is also an important way to create the next generation in GA for genes diversity. The algorithm generates the mutation children from the parents’ genes by choosing a random number from the Gaussian distribution (see [76]). An example of mutation is demonstrated in Figure 9.

#### 4.4.4. Evaluation and Iteration

The current generation population is evaluated by the fitness function. The iteration of creating new generations is terminated once the fitness performance meets the requirement, or the iteration number reaches the maximum iteration limits.

## 5. Case Study

**Figure 10:**
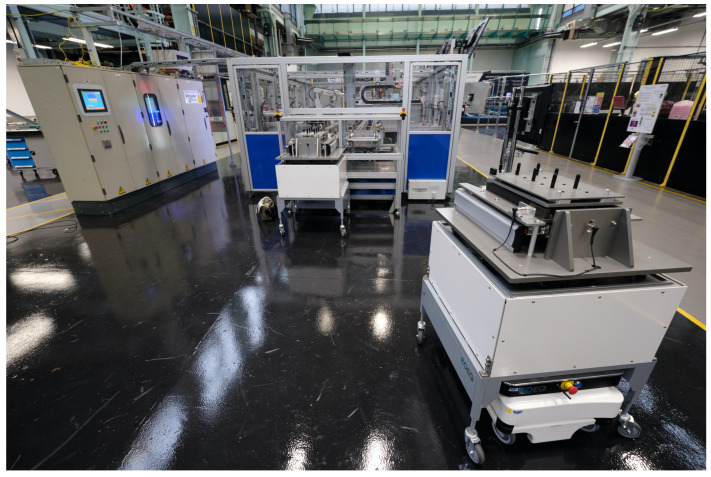
An example material transportation within the IML rig: An AGV is carrying battery cells to the Legacy Loop Assembly Machine in the Stage One where battery modules are assembled.

The case study is implemented in the IML demonstrator at the University of Warwick. IML is designed as a discrete-part automation system assembling battery-packs for electric vehicles. The battery assembly process includes customised battery packs from a single battery cell, such as: 18650, 26650. IML deploys a variety of legacy and agile systems—a traditional conveyor based system represents traditional cellular manufacturing practice [77], while autonomous stations, connected by AGVs for battery pack welding and vision based inspection, represent an Industry 4.0 based example of responsive manufacturing. Figure 10 shows a section of the IML rig. The case study shows the optimisation methodology to improve the manufacturing performance of battery assembly process.

### 5.1. Overview of the Experiments

The case study describes a battery assembly process based on the IML demonstrator prototype. The production is modelled and simulated via a DES model. The model’s input values are fed by the proposed optimisation model, and the predicted of KPI values are served as feedback, thereby indicating a closed-loop system for improving the battery assemble process JIT performance. The assembly system is separated into four stages, including the Legacy Loop Assembly stage, Welding Stage, Inspection Stage, and Packing Stage. Cycle time and machine tool changing time are predefined from the historical data from the IML demonstrator. In addition, AGV speed, AGV charging time, and AGV running time are assigned as AGV attributes based on the MiR100 servicing in the IML demonstrator. In stage One and Three, the battery cell insertion and nut assembly operations are carried out, respectively. These operations require raw materials such as module baskets and nuts. Stages Two and Four perform welding and inspection processes, respectively. Materials between each stage are delivered and collected by AGVs. Customer orders are recorded by a web-based products order system. Once an order is issued, this information will be published to the OPC-UA server. In the OPC-UA server, the data from IML demonstrator rig, e.g., PLC registers and I/O, buffer sensors status, and product RFIDs information are recorded. When an order arrives, the decision support system optimises the arrival product sequence and AGV schedules. Once the system finishes the optimisation process, it will broadcast a list of optimised solutions and corresponding production KPIs on the system HMI which can be accessed by system managers or operators to manually choose the proper solution. As soon as a solution is selected, the decision support system will pass this information to the MES application, written in C language, to assign the defined task to corresponding working stations and MiR fleet manager.

In the experiment, 30 jobs are designed to be processed. These jobs are separated into 20 different categories. Each job has four processes, and each stage of the process has four parallel machines. In the experiments, the simulation run-time is set as 25,000 s. Please note that the simulation is forced to terminate when the time runs out, and KPI values will not be recorded. The DES model and embedded GA-based optimisation algorithm are concurrently run to find the Pareto-optimal design space. There are two stopping criteria for GA: (*i*) stop by reaching the maximum number of generations (1000) and (*ii*) stop by max stall generations (30). Moreover, the production target time is set as 4 h per shift, including 3.75 h (135,000 s) processing time and 0.25-h break time. The input parameters are separated into two parts: the first four indicate non-integer parameters, i.e., weights of each machine stage, which the arrival parts sequence can be derived from; the rest of 120 integer values are AGV distribution rules for every arriving part. They are converted as arrival parts attributions and transfer to the WITNESS simulation model. Moreover, the production KPIs are collected as outputs to the optimisation model.

To evaluate the re-scheduling performance of the proposed framework, machine breakdown scenarios were also introduced. In those experiments, after 4000 s of overall process time, two machines were intentionally shut down and process stops. The SAMS is expected to detect the abnormality by solely monitoring the PLC status and resources cycle times. Once the fault information is received by the SAMS, the re-schedule procedure starts. This process involves updating the DES model, executing the simulation for the remaining tasks, and re-allocates the tasks between system resources as soon as a re-schedule among the solution set is approved. After 40 min (2400 s) of hypothetical repair time, the broken machines were back to operation. The SAMS initiates a second re-scheduling process and feeds the new set of solutions into MES application for the approval.

The following assumptions were made during the experiments:

- The shop-floor layout and AGV routing paths were fixed.
- Charging threshold for AGV is set at 20%. If the battery level is lower than 20%, the AGV needs to park at the charging station for re-charge. When AGV battery is fully charged, it will be ready for the new task.

In the experiments, the initial machine parameters, including: setup time and cycle time, and AGV average speeds, non-stop travelling time, and charging time, are collected from the shop-floor through IoT-enabled data collection devices. For instance, the RFID tags are used for tracking battery pallets and calculating the commuting time between each station, energy monitors are attached at each workstation to collect the energy consumption, and PLC function blocks are programmed to calculate machines and robots cycle times. In addition, these data are fed into the OPC UA server through Modbus TCP/IP protocol. The DES model is implemented in WITNESS software, and the optimisation engine is achieved through MATLAB programming language. The experiments presented here are deployed on a PC with Intel(R) Xeon(R) with a 32 GB RAM and I7 8-core 3.8 GHz processors. Please note that the average time for each process simulation within the DES environment is recorded as 5 ± 1 s. Based on the experiments, the GA converges around 200 simulation runs. This indicates a total scheduling optimisation run about 11 ± 3 min (including decision-support and communication with MES). Please note that this is based on the experiments we carried out with simple machine breakdown scenarios at the IML.

**Figure 11:**
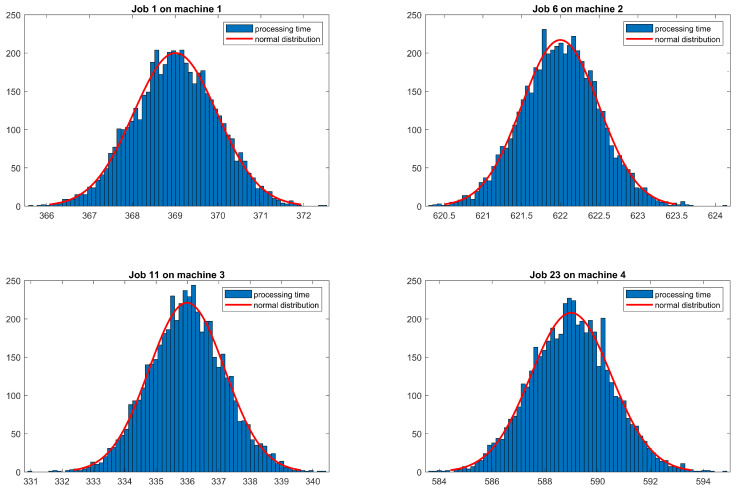
Example of job processing time distribution.

Figure 11 shows the histograms of the job processing times for selected machine operations. A uniform normal distribution is selected to represent these job processing times based on the data stored in the time-series database:

(11) $$\begin{array}{c} PT∼N(\mu ,\;\sigma^{2})\;. \end{array}$$

where the μ means the average processing time(PT), and σ means the standard deviation of these collected processing time. The μ and σ changes with different jobs. In this case study, both parameters (μ,σ) for each job are analysed, and then updated in the DES software. Please note that the time-series database includes more than 5000 sampling points for each operation.

### 5.2. Results

**Figure 12:**
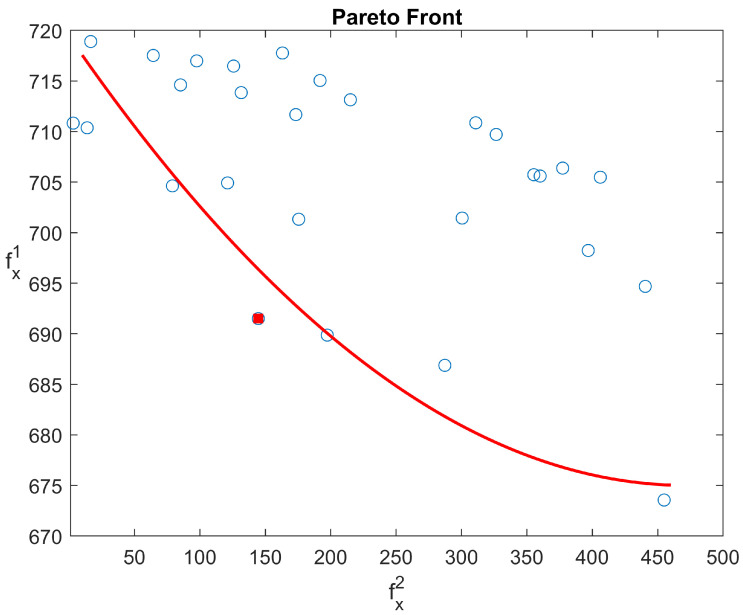
The Pareto Front.

The result for static job scheduling problem is given in Figure 12, showing the relationship of the tardiness of the material delivery and the average AGV energy consumption. It has been found that AGV energy consumption and JIT material delivery performance are two conflicting outputs. Hence, an optimal scheduling strategy is required. In this research, the relative Euler distance method is chosen to find the near-optimal solutions for AGV and machine jobs scheduling:

(12) $$\begin{array}{c} Dis\left(f_{1},f_{2}\right)={\left(\frac{\left(f_{x}^{1}-f_{min}^{1}\right)}{\left(f_{max}^{1}-f_{min}^{1}\right)}\right)}^{2}+{\left(\frac{\left(f_{x}^{2}-f_{min}^{2}\right)}{\left(f_{max}^{2}-f_{min}^{2}\right)}\right)}^{2} \end{array}$$

**Figure 13:**
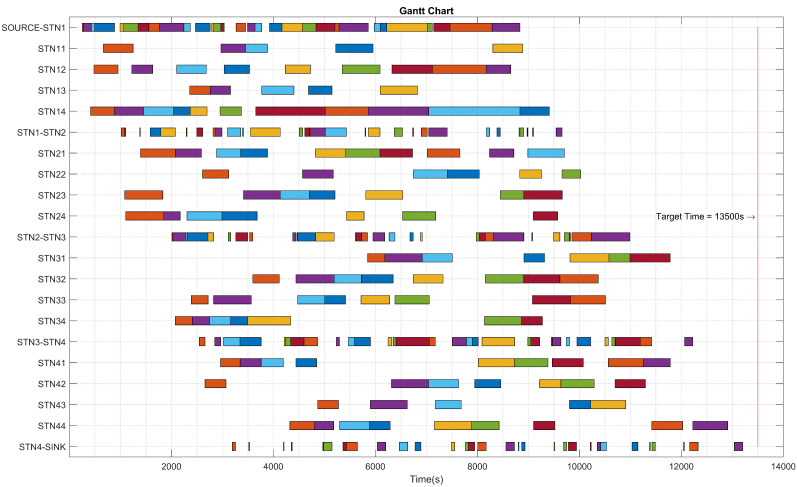
Gantt Chart (Normal/planned events).

In the equation given above, the $Dis(f_{1},f_{2})$ represents the Euler distance between two objective functions, and the minimum value is considered as the best solution in this paper. $f_{max}^{1}$ and $f_{min}^{1}$ represent the minimum and maximum value of 1st objective function, respectively, and $f_{max}^{2}$ and $f_{min}^{2}$ represent the minimum and maximum value of 2nd objective function. Once the solution parameters for the Euler distance are set, the best solution for machine jobs schedule and AGV distribution rules can be attained. In this way, multiple solutions can be provided based on different KPIs requirements, including AGV blocking time, machines utilisation balance, and parts waiting time in the buffer, etc. Figure 13 depicts the Gantt chart for the best solution including both machine and AGV schedules for static job scheduling experiments.

To evaluate the efficiency of the proposed system, its performance is compared with a static First-In-First-Out (FIFO) and five Shortest Processing Time (SPT) based dispatching methods. In the FIFO-based dispatching approach, the first arrival product is delivered to the nearest machine, and be processed first. On the other hand, SPT-based method prioritises the product with the shortest processing time. Here, five different SPT-based scheduling rules, i.e., the SPT based on the cycle-time for each stage and the SPT based on the overall product cycle-time. The performance comparison is given in Table 3. According to the results, a large tardiness improvement is recorded for the proposed approach. It is also noted that a slight increase in AGV energy consumption (EC) performance is achieved.

Two production scenarios with manufacturing disruptions are also set up to evaluate the re-scheduling capability of the proposed approach. In these scenarios, the fourth machines in Stage 2 and Stage 3 are intentionally broken down. The breakdown is set from 4000 s to 6400 s, lasting for 40 min. Meanwhile, the re-scheduling strategies are generated by the SAMS to meet the JIT requirements with an acceptable AGV energy consumption rate. The results (Table 3) showed that the SPT and FIFO-based methods are unable to handle manufacturing interruptions, although they are capable of providing acceptable performance under normal operational conditions. The proposed approach is able to effectively re-schedule AGV and machine schedules subjected to production abnormalities, and provides a significantly better tardiness performance. Please note that all methods have similar results for AGV energy consumption rates.

### 5.3. Discussion

With the recent advancement in the Industry 4.0 systems and technologies, the decision-support systems became a vital enabler in ensuring global competitiveness of manufacturing enterprises. In the related literature, there are a few-number of works involving the simulation-based decision-support systems within the context of manufacturing systems engineering. Some examples include: [78,79,80,81]. Contrary to exact methods, the simulation-based approaches provide timely decisions due to reduced computational complexity. However, these methods are often criticised due to accuracy problems [82]. In this research, the SAMS architecture is modified to overcome this challenge. To minimise the prediction errors of the static DES models used within the SAMS, IoT-enabled historical data are streamlined into the DES models to enhance their prediction capabilities. In addition to this, an evolutionary optimisation algorithm (i.e., GA) is employed in a multi-objective optimisation problem to deal with the scheduling complexity while avoiding getting trapped in the local minima. The interoperability of the proposed system is demonstrated using OPC-UA industrial M2M communication protocol. Moreover, the decision-support capabilities of the approach are demonstrated on case studies where a set of near-optimal re-scheduling solutions are promptly provided to shop-floor managers upon the event of manufacturing disruption via a human–machine interface. The results showed that the proposed approach can help to improve the performance of the system in terms of just-in-time delivery performance, the average utilisation of the system resources, average queue times, and energy efficiency of AGV transportation.

The Lanner’s WITNESS DES software provides an object-oriented modelling approach for AGV material transportation. The model has a pre-defined AGV routing topology that ensures that AGVs do not collide against each other. A deadlock consists of a model state in which the AGVs are simultaneously waiting for any other AGV to perform a task and no AGV can change its current state. Effectively, this locks the model, and prevents the completion of the simulation run. During the initial modelling stages, which involves identifying potential issues, we observed that the possibility of deadlock occurrence when an AGV tries to access to the storage locations. This is because the access to the storage location was done using a bi-directional path with a single unit capacity. This allows only one AGV to cross this path at any time with another AGV waiting on the other end of the path and there is no space for the first AGV to exit. It is important to note that this type of deadlock should be avoided during the simulation. To prevent this issue, we introduced two unidirectional paths across the routing topology. This, in fact, can be considered as a crude simplification of the real system. However, since the IML demonstrator under consideration has a very low number of AGVs, this situation rarely occurs in the real system. Therefore, it is assumed that the addition of two unidirectional paths in the simulation model has a negligible impact on the results. Please note that, for systems with complex layouts and/or a high number of AGVs, more sophisticated deadlock prevention algorithms and mechanisms should be employed. Some examples include: [83,84,85,86,87].

The proposed approach, however, has certain limitations that need to be addressed. Firstly, in its current form, the SAMS operates with limited data. As future work, to fully exploit the advantages of the concept of Big Data Analytics, more IoT-enabled data will be streamlined into the SAMS and a complex event processing engine will be employed to process those streams. This will provide a better understanding of the relationships among various shop-floor activities and will help to improve the predictive analytics capabilities of the approach. Another important limitation is the prediction errors arising due to the real-time behaviours of AGVs. The proposed SAMS provides a set of scheduling alternatives based on the simulation optimisation results. The selected schedule and corresponding AGV job assignments are then fed to the MES and further MiR fleet manager. The MiR fleet manager is an industrial control system for AGVs providing a collision-free routing with shortest travel times. The fleet manager assigns tasks to AGVs depending on their location, energy levels, etc. This manager has an in-built traffic control mechanism offering the coordination of critical zones with multiple robot intersections and hence providing a collision free routing. Additionally, MiR AGVs have collision sensors and in-built cartographer SLAM algorithms to prevent any real-time collision issues. AGVs can autonomously decide and manoeuvre outside of their pre-defined path to avoid any type of collisions. It is important to note that there might be differences in the AGV path since the WITNESS models have pre-defined routes unlike the MiR fleet manager. In the experiments, we observed a difference between completion time of shop-floor jobs and DES simulation results (up to 7.1%) because of logistics uncertainties. This limitation of the SAMS will be addressed as future work by employing a better information-mirroring mechanism between cyber and physical domains. The graphical user interface used in the SAMS decision-support system only broadcasts a list of solutions to be selected on the HMI screens. As future work, a new dashboard with varying visualisation options will be developed to provide a better decision-support to shop-floor decision-makers. Lastly, the communication between the proposed systems and MES and Enterprise Resource Planning (ERP) systems will be enhanced using web-services to provide a more industry-ready deployable solution.

## 6. Conclusions

In this paper, a decision-support system capable of providing multiple scheduling solutions as a response to manufacturing disruptions was introduced. The system uses IoT-enabled production data to enhance the accuracy of the digital replica of the FMS under consideration. In the event of a manufacturing disruption, the system automatically detects the production anomaly and releases a set of re-scheduling strategies aiming to satisfy both maximised just-in-time delivery performance and minimised AGV energy consumption on time. The system was tested on a real industrial case study, and the results showed that the system is helpful to managers for the decision-making at the operational level.

## Figures and Tables

**Table 1:** A summary of the related literature review.

Type	Examples	Strengths	Weaknesses
Offline	[4,8,20,21,22,23,24,25,26,27,28,29,30,31,44,45,46,47,48,49,50,51,52,53,54,55,56,57,57]	Handles scheduling complexity	Inflexibility
scheduling		Low CPU overloads	Deterministic behaviours
			Requires task arrival information
			Subjected to a limited execution time
Online	[32,33,34,35,37,38,39,41]	Handles unpredictable workloads	Reduced utilisation of resources
scheduling			CPU overloads are harder to detect

**Table 2:** Notations.

Notation	Description
Sets	
*S*	Set of stations
*T*	Set of production jobs
$N^{AGV}$	Set of AGVs
*W*	Set of workstages
$S_{w}$	Number of stations in stage *w*
Indices	
*s*	Index of station, s∈{1,2,...,S}
*t*	Index of production job, t∈{1,2,...,T}
*n*	Index of AGV, $n\in \{1,2,...,N^{AGV}\}$
*w*	Index of workstage, w∈{1,2,...,W}
$s_{w}$	Index of station in stage *w*, $s_{w}\in \left\{1,2,...,S_{w}\right\}$
Parameters	
$Q_{n_{o}}$	The weight of no load AGV *n*
$Q_{ijnt}$	The weight of AGV *n* loaded, when travelling between station *i* and *j* for job *t*
α	Earliness cost penalty coefficient
β	Lateness cost penalty coefficient
$PT_{tsw}$	Processing time of job *t* allocated to *s* in stage *w*
$d_{t}$	Due date of job *t*
$C_{t}$	Completion date of job *t*
$S_{tsw}$	Starting time of job *t* at station *s* in stage *w*
$D_{tsw}$	Completion time of job *t* at station *s* in stage *w*
$dis_{ij}$	Distance between station *i* and *j*, also, i≠j
$r_{t}$	Release time of the job *t* into the system
Decision Variables	
$M_{tsw}$	1 if machine $s_{w}$ working on job *t*, else 0
$X_{ijnt}$	1 if AGV *n* travels between station *i* and *j* for job *t*, else 0

**Table 3:** Comparison of the implemented scheduling approaches.

Solutions	Normal Events		Two Machines Breakdown	
	Tardiness	EC	Tardiness	EC
Proposed Scheduling	300.4484 (Earliness)	701.4404	218.6914 (Earliness)	704.9327
FIFO Scheduling	1575.7169 (Earliness)	577.4241	4657.8487 (Lateness)	701.1848
SPT based on 1st Stage	1103.9 (Earliness)	585.7565	14,968 (Lateness)	997.0096
SPT based on 2nd Stage	679.377 (Earliness)	607.6007	13,708 (Lateness)	923.2472
SPT based on 3rd Stage	1223.6 (Earliness)	612.8167	9681.7 (Lateness)	833.9795
SPT based on 4th Stage	1179.2 (Earliness)	613.1612	15,750 (Lateness)	1150.4
SPT based on overall Stage	1710.9 (Earliness)	576.6980	13,956 (Lateness)	944.900
